# Evolving e-taxonomy

**DOI:** 10.1186/1471-2148-9-141

**Published:** 2009-06-25

**Authors:** Hans Zauner

**Affiliations:** 1 BioMed Central, 236 Gray's Inn Road, London, WC1X 8HL

When Carl von Linné published his work *Systema Naturae *[1] he laid the foundation for the literature that builds the science of taxonomy. For scientists today, permanent access to the original species descriptions published since von Linné's time is of crucial importance. Every new generation of taxonomists builds on the work of their predecessors and adds to the mind-boggling enterprise of describing the earth's entire biodiversity at the level of single species. Following 250 years of taxonomy, around 1.8 million species of animals, plants and microorganisms are known to science, whilst the total number of species might be between 10 and 100 million [2]. There is no time to waste in filling the knowledge gap, with biodiversity under threat and the reality that many species die out before we even know about their existence.

A new breed of scientists, 'cybertaxonomists' and 'biodiversity informaticians', are now setting up a framework to deal with the bank of data that has been collected in the last 250 years and to help share what will be gathered in the future. The common aim is not only to have an easily accessible record of species descriptions, but to cross-link different types of biodiversity data, connecting for example biogeography, climate data and phylogenetic information and also to incorporate new technologies like GPS tracking and advanced imaging techniques. Several web-based initiatives are devoted to this research program, each with a slightly different goal. For example, the vision of *Encyclopedia of Life (EoL) *[3], as expressed by Edward O. Wilson, is:

"*"Imagine an electronic page for each species of organism on Earth, available everywhere by single access on command*."

Over a period of five years, EoL plans to generate a million species pages and to digitize biodiversity literature.

Another example of a project aimed at stitching together information technology and biodiversity research is the *Global Biodiversity Information Facility *(GBIF) [4]. GBIF aims to provide an informatic architecture to make primary biodiversity data accessible to everyone. These and similar projects are driven by the spirit of data sharing that has been so beneficial throughout the history of science. Such initiatives bring together data from various fields, but at the heart of all these efforts are individual species, some of them first described and named ages ago, most of them yet to be known. The critical challenge that taxonomists face is to maintain continuity in their century-spanning mission, while adapting to the revolution in information technology.

To avoid uncertainty and provide rules for the naming of new animal taxa, the International Committee for Zoological Nomenclature (ICZN) [5] has provided elaborate guidance, for example in deciding which name has priority should more than one name appear in the literature for the same species. Most scientists agree that the current version of this code needs to be revised to make taxonomy fit for the digital future. Access to sometimes very specialized primary literature, often aimed at an audience of a few experts around the world, is of pivotal importance in taxonomic research and electronic access via web-based resources is increasingly becoming the most efficient way to find and retrieve these articles. Unfortunately, the current version of the ICZN code makes life needlessly complex for online journals that publish species descriptions (such as *BMC Biology*, *BMC Evolutionary Biology *and *Frontiers in Zoology*) by requiring the production of printed copies.

A positive development is a proposed amendment to the code, which would allow animal species descriptions and other nomenclatural acts to be published in electronic format, provided a number of conditions are met to ensure long-term accessibility [6]. Of course, not everything that is posted on the Internet is assured permanency and it therefore makes sense that the proposed amendments of the code insist on some prudent criteria. For example, copies of the PDF files of published articles should be deposited in a number of repositories that are independent of the publisher (something which is already the case for all BioMed Central articles). A separate proposal is that the validity of new species names might be determined not by print or electronic publication, but by the registration of the name with an official registry, Zoobank [7], which already exists in experimental form. Upon registration, each new species would get a unique Life Science Identifier (LSID), which would provide a standard reference analogous to the GenBank accession number assigned to new DNA sequences.

The community of zoological taxonomists is now discussing these suggestions and a variety of views and commentary regarding the merits of electronic publication and open access for species descriptions have been recently published in an issue of the Bulletin of Zoological Nomenclature [8]. BioMed Central has been an active participant in this debate, and looks forward eagerly to the next update to the ICZN code.

Finally, although the issue of long-term availability of electronic publications is distinct from that of Open Access, it is hard to see how a cross-linked, integrated network of online biodiversity resources can achieve its full potential if access to key primary sources, such as species descriptions, is restricted. It is therefore unsurprising that many leading researchers in the new fields of cybertaxonomy and biodiversity informatics are passionate supporters of Open Access publishing. In a recent article in *BMC Research Notes*, Donat Agosti and Willi Egloff describe an exciting approach to improve access to taxonomic information by providing 'semantically enhanced' documents that are machine-readable and that are openly shared and cross-linked to other resources [9]. At BioMed Central, we are doing all we can to encourage and facilitate these new developments, as evidenced by our involvement in the recently launched GBIF Task Group on a Data Publishing Framework [10] and we continue to work closely with our authors, referees, and Editorial Boards to develop publishing services that meet the needs of researchers working on biodiversity and taxonomy.
